# The immediate effects of mobilization with movement on shoulder range of motion and pain in patients with rotator cuff-related shoulder pain: A randomized controlled trial (Evolution Trial)^☆^^☆☆^

**DOI:** 10.1016/j.bjpt.2024.101145

**Published:** 2024-11-20

**Authors:** Sizhong Wang, Jiaxu Zeng, Ramakrishnan Mani, Cathy Mary Chapple, Daniel Cury Ribeiro

**Affiliations:** a Centre for Health, Activity and Rehabilitation Research (CHARR), School of Physiotherapy, University of Otago, Dunedin, Otago, New Zealand; b Division of Physiotherapy, Department of Health Sciences, College of Health, Medicine and Life Sciences, Brunel University London, Uxbridge, London, United Kingdom; c Centre for Physical Activity in Health and Disease (CPAHD), Brunel University London, Uxbridge, London, United Kingdom; d Department of Preventive and Social Medicine, Dunedin School of Medicine, Division of Health Sciences, University of Otago, Dunedin, Otago, New Zealand.; e Curtin School of Allied Health, Faculty of Health Sciences, Curtin University, Perth, WA, Australia

**Keywords:** Angular onset of pain, Mobilization with movement, Pain intensity during movement, Rotator cuff-related shoulder pain, Shoulder

## Abstract

**Background:**

Mobilization with movement (MWM) is commonly used to treat patients with rotator cuff-related shoulder pain (RCRSP). However, the evidence supporting MWM efficacy for improving range of motion (ROM) and pain in patients with RCRSP is limited.

**Objectives:**

To assess the immediate effects of MWM on the angular onset of pain in patients with RCRSP.

**Methods:**

Sixty-three participants with RCRSP were randomized to receive 3 sets of 10 repetitions of MWM or sham MWM with a minute rest between each set. The angular onset of pain (primary outcome), and pain intensity at rest and during shoulder abduction to the onset of pain were measured at baseline, and after receiving the 1^st^ and 3^rd^ sets of 10 repetitions of interventions. Other secondary outcomes were measured at baseline and after receiving 3 sets of 10 repetitions of interventions or 1, 2, 3, 5, and 7 days after interventions.

**Results:**

Compared with the sham MWM group, the MWM group had an additional improvement of 6.5° (95% CI -0.9, 13.9) and 13.7° (95% CI 6.3, 21.1) (from baseline) after receiving the 1^st^ and 3^rd^ sets of 10 repetitions of interventions, respectively.

**Conclusion:**

MWM improves the angular onset of pain after 3 sets of 10 repetitions of MWM in patients with RCRSP. This study provides preliminary support for the use of MWM in treating patients with RCRSP and provides some guidance for clinicians to decide the MWM dosage to be used in clinical practice.

## Highlights

• 1 set of 10 repetitions of MWM caused small improvements in angular onset of pain.
• 3 sets of 10 repetitions of MWM improved angular onset of pain.
• Those effects were observed with a group of patients who had predominantly chronic shoulder pain.

## Introduction

Mobilization with movement (MWM) is a manual therapy technique that aims to restore full range of pain-free movement and is commonly used for treating patients with rotator cuff-related shoulder pain (RCRSP).^1^^,^^2^ Clinicians implement MWM in clinical practice with the aim of immediately improving pain-free range of motion (ROM) and reducing pain experienced during movement. According to classic textbooks and as per clinical practice, MWM should only be applied if the patient reports an immediate improvement in ROM or pain when the technique is first applied.^1^^,^^2^ Despite its common use, the effect of MWM for improving ROM in patients with RCRSP is uncertain,^3^ and the quality of evidence is low.^4^

There are many uncertainties regarding the effects of MWM on ROM and pain. The isolated effect of MWM on the angular onset of pain in patients with RCRSP remains controversial.^5^, ^6^, ^7^, ^8^ It is unclear whether MWM has any effect on pain modulation in patients with RCRSP.^8^^,^^9^ It is uncertain how many sets and repetitions of MWM patients should receive in a session.^2^ The majority of studies testing the effect of MWM on patients with RCRSP adopted a dosage of 3 sets of 10 repetitions.^5^^,^^6^^,^^8^^,^^10^, ^11^, ^12^, ^13^, ^14^, ^15^ According to the literature and anecdotal evidence from clinical practice, clinicians may use a lower number of sets or repetitions of MWM. No previous studies explored the immediate effect of different dosages of MWM (e.g., 3 sets of 10 repetitions versus 1 set of 10 repetitions) on ROM or pain in patients with RCRSP.

The inconsistent findings from previous trials are likely influenced by limitations presented by those trials (e.g., small sample size, the use of crossover study design or single arm pilot design, heterogeneity of dosage of MWM tested, and the use of different types of quantitative sensory testing),^8^^,^^9^ or different dosages of MWM (i.e., numbers of sets, repetitions, or treatment sessions)^16^ tested within those trials. In addition, not all trials have used the criteria of immediate positive response to this techinique when assessing the effect of MWM on pain or ROM. There is a need for high-quality trials to assess the immediate, short-term, and long-term effects of MWM interventions.^4^

The primary objective of this study was to (1) assess the immediate effect of MWM on the angular onset of pain (in degrees) during shoulder abduction in patients with RCRSP after receiving the 1^st^ set and 3^rd^ set of 10 repetitions of intervention. The secondary objectives were to (2) assess the immediate effects of MWM on pain intensity during shoulder abduction (to the onset of pain and during maximum range), pain intensity at rest, maximum ROM during shoulder abduction, pressure pain threshold (PPT), mechanical temporal summation (MTS) scores, and improvement of the global rating of change scale (GROC) after receiving the 1^st^ set or 3^rd^ set of 10 repetitions of intervention and 3 days after the intervention, (3) assess the incremental effect of 2 sets of 10 repetitions of the MWM intervention on the angular onset of pain, pain intensity during shoulder abduction to the onset of pain, and pain intensity at rest after receiving the 1^st^ set of 10 repetitions of intervention, and (4) explore the self-report changes in pain intensity and interference over time (days 1, 3, 5, and 7) after receiving 3 sets of 10 repetitions of intervention.

## Methods

### Design

This was a single centre, participant- and assessor-blinded, randomized, and sham-controlled trial. All outcome measures were assessed by a researcher (SW) who was blinded to randomization. All participants provided informed written consent prior to taking part in the study. When writing this manuscript, we followed the Consolidated Standards of Reporting Trials Statement.^17^ The trial protocol^18^ has been published previously.

### Study setting

The study was conducted at the Centre for Health, Activity and Rehabilitation Research, School of Physiotherapy, University of Otago.

#### Ethics approval

The University of Otago Ethics Committee approved this study (Ref. H21/117).

### Participants

We recruited participants with RCRSP from 29 March to 4 August 2022. Participants were recruited using periodic advertisements on social media (e.g., Facebook) and announcements through email to staff and students from the university in which this study was conducted. The researcher (SW) screened participants following the British Elbow and Shoulder Society (BESS) guidelines.^19^ The inclusion criteria for participants were: (1) aged from 18 to 75 years,^20^ (2) present with a painful arc of movement during shoulder abduction; or pain on resisted lateral rotation or abduction; or positive Jobe's test,^19^ (3) respond positively to the application of the shoulder MWM using sustained posterolateral glide, and (4) able to provide written informed consent. We opted to include only participants who responded positively to MWM to ensure we followed recommendations from the literature on this topic,^1^^,^^2^^,^^15^ as well as current clinical practice. Clinicians should not apply MWM technique to patients who do not respond positively to an initial application.^1^^,^^2^ By adopting this inclusion criteria, we increased the external validity of our findings.

Participants with any of the following conditions were excluded: (1) signs or symptoms suggesting acute rotator cuff tear or massive rotator cuff tears,^21^ (2) history of shoulder or cervical surgery in the past six months,^22^, ^23^, ^24^ (3) history of corticosteroid injection on the affected shoulder in the last six weeks,^20^ (4) other shoulder disorders, (5) symptoms of paraesthesia in the upper extremity, (6) neurological disease affecting shoulder pain and/or function, and (7) systemic inflammation or disease, or tumour. Further information on the screening process is available in the study protocol.^18^

### Procedures

**Fig. 1:**
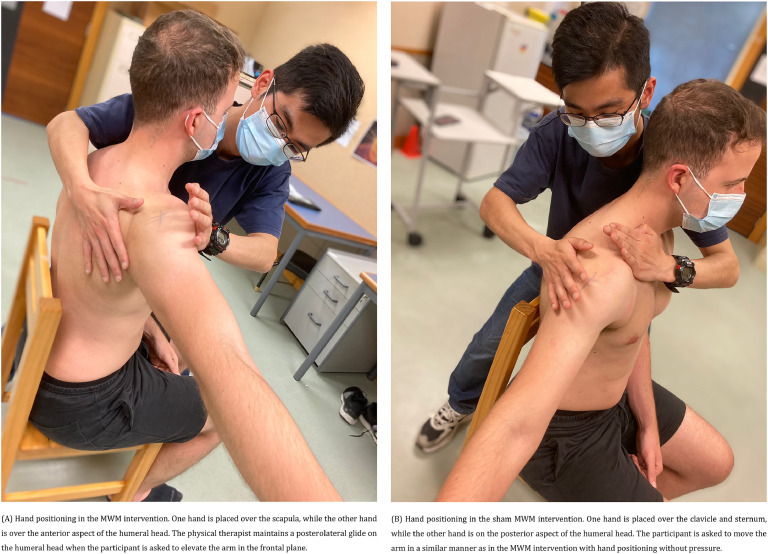
Mobilization with movement (MWM) and sham MWM interventions. (A) Hand positioning in the MWM intervention. One hand is placed over the scapula, while the other hand is over the anterior aspect of the humeral head. The physical therapist maintains a posterolateral glide on the humeral head when the participant is asked to elevate the arm in the frontal plane. (B) Hand positioning in the sham MWM intervention. One hand is placed over the clavicle and sternum, while the other hand is on the posterior aspect of the humeral head. The participant is asked to move the arm in a similar manner as in the MWM intervention with hand positioning without pressure.

The data collection processes are presented in the Supplementary material: Figure S1. The included participants attended two sessions at least two days apart and within one week. In the first session, participants were screened by the researcher (SW), and they provided their demographic information as well as completed the following validated questionnaires: patient-specific functional scale (PSFS),^25^ shoulder pain and disability index (SPADI),^26^ depression, anxiety, and stress scale (DASS-21),^27^ pain catastrophizing scale (PCS),^28^^,^^29^ shoulder specific fear-avoidance beliefs questionnaire (FABQ),^30^ pain self-efficacy questionnaire (PSEQ-2),^31^ and EuroQol-5D five-level version of EQ-5D (EQ-5D-5 L).^32^ In their second visit, all participants completed the following validated questionnaires: Brief pain inventory-short form (BPI-SF),^33^^,^^34^ and expectation for treatment scale (ETS).^35^ After baseline outcome measures, all participants were then randomly allocated to either the MWM or sham MWM group with an allocation ratio of 1:1 using varying block sizes. Randomization lists were generated by the study statistician (JZ) using R Software.^36^ Interventions were delivered by three physical therapists according to the randomization sequence sealed in an envelope. All three physical therapists (DCR, GF, and MR) have experience in the management of patients with musculoskeletal disorders and familiar with the MWM technique. The physical therapist informed participants that the intervention, i.e., MWM (Fig. 1A) or sham MWM (Fig. 1B), should be pain-free and asked participants to inform them if symptoms were aggravated during the procedure. The volume of intervention consists of 3 sets of 10 repetitions of MWM or sham MWM with an interval of 60 s between sets. All participants and outcome assessor were blinded to the interventions. The angular onset of pain (primary outcome), pain intensity at rest, and pain intensity during shoulder abduction to the onset of pain were assessed at baseline, and immediately after 1^st^ and 3^rd^ sets of interventions. Other secondary outcomes were measured at baseline and after receiving the 3^rd^ set of 10 repetitions of interventions or 1, 2, 3, 5, and 7 days after interventions. All outcome measures were assessed by one researcher (SW). Detailed information of the study procedure is available on the trial protocol.

### Primary outcome measure

The primary outcome was the angular onset of pain, measured using a digital inclinometer and expressed in degrees. If participants presented no pain at rest, we recorded participants’ angular onset of pain when participants started to feel pain during active shoulder abduction. If participants presented pain at rest, the angular onset of pain was defined as the ROM in which participants felt their shoulder pain started to increase during active shoulder abduction.^37^ We opted for this outcome measure because clinicians expect MWM to immediately improve pain-free ROM (i.e., angular onset of pain) when they use this technique in clinical practice.

### Secondary outcomes measure

The secondary outcomes were pain intensity at rest, pain intensity during shoulder abduction to the onset of pain, pain intensity during shoulder abduction to the maximum range, maximum ROM, PPT, MTS, GROC, harm, and BPI-SF. A detailed description of the primary and secondary outcome measures is presented in the published protocol.

### Data analysis

Based on a previous study conducted by our group,^7^ assuming a standard deviation (SD) of 19°, a sample size of 28 participants per group was required to detect a 14.5° difference between the two intervention arms, with a power of 80% and a significance level of 5%. Given the short time required for participants to be involved in the study, the chance for dropout was very small. Hence, we assumed a 5% dropout rate. The minimum sample size required was 30 participants per group. The sample size was calculated using G*Power (Version 3.1, University of Kiel, Germany).

A linear mixed effects model with a random intercept was used to compare the changes in outcome measures (from baseline to the time after receiving the 1^st^ set and 3^rd^ set of interventions, respectively) between the MWM and sham MWM groups (Objectives 1 and 2). The model included time, intervention and an interaction between time and intervention as covariates. All time points (baseline, after the 1^st^ set of interventions, and after the 3^rd^ set of interventions) were retained as part of the outcome variable. An independent *t*-test was used to compare the GROC scores between the MWM and Sham MWM groups after 3 sets of interventions and 3 days after receiving the interventions, respectively (Objective 2). We estimated the between-group difference in changes in the outcome measures from time 1 (after receiving the 1^st^ set) to time 2 (after receiving the 3^rd^ set) (Objective 3). We reported the mean difference in outcomes and their respective 95% confidence intervals. We assessed the difference in changes in BPI-SF over time (days 1, 3, 5, and 7 after intervention) between the MWM and sham MWM interventions (Objective 4). We adopted an intention-to-treat (ITT) analysis when conducting those analyses. All statistical analyses were performed using Stata 17.0 software (Stata Corporation, College Station, TX, USA).^38^

## Results

### Characteristics of participants

**Fig. 2:**
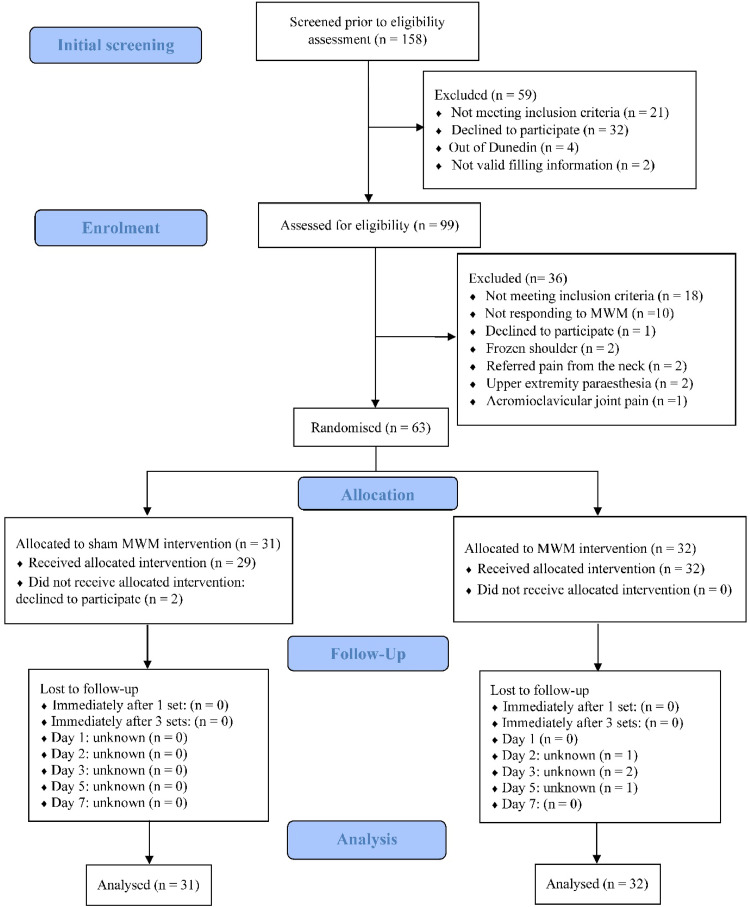
Flowchart of the participant through the trial. MWM, mobilization with movement.

One-hundred-fifty-eight participants were screened initially for study eligibility, and 63 participants met the criteria and agreed to participate in this study (Fig. 2). They were randomly allocated to receive either sham MWM (*n* = 31) or MWM (*n* = 32). Demographic and clinical characteristics of participants are presented in Table 1.

**Table 1:** Characteristics of participants.

Variables	Sham MWM	MWM	Total
	(*n* = 31)	(*n* = 32)	(*n* = 63)
Sex, n (%)			
Male	15 (48)	16 (50)	31 (49)
Age (years)	50.1 (15.4)	45.3 (16.0)	47.6 (16.0)
Weight (kg)	82.8 (18.5)	80.8 (18.3)	81.8 (18.3)
Height (cm)	170.2 (10.8)	169.7 (9.2)	170.0 (10.0)
Body Mass Index (kg/m^2^)	28.4 (5.4)	28.1 (6.9)	28.3 (6.1)
Employment, n (%)			
Employed full-time	21 (67.7)	17 (53.1)	38 (60.3)
Employed part-time	3 (9.7)	4 (12.5)	7 (11.1)
Self-employed	3 (9.7)	2 (6.3)	5 (7.9)
Unemployed	0 (0.0)	1 (3.1)	1 (1.6)
Retired	3 (9.7)	3 (9.4)	6 (9.5)
Student	1 (3.2)	5 (15.6)	6 (9.5)
Ethnicity*, n (%)			
European	31 (100.0)	27 (84.4)	58 (92.1)
Māori	3 (9.7)	4 (12.5)	7 (11.1)
Pacific	1 (3.2)	0 (0.0)	1 (1.6)
Asian	1 (3.2)	3 (9.4)	4 (6.3)
Other	0 (0.0)	1 (3.1)	1 (1.6)
Unknown	0 (0.0)	1 (3.1)	1 (1.6)
Education, n (%)			
No qualifications	1 (3.2)	1 (3.1)	2 (3.2)
Secondary school	7 (22.6)	5 (15.6)	12 (19.1)
Post-secondary	11 (35.5)	9 (28.1)	20 (31.8)
University degree or above	12 (38.7)	17 (55.1)	29 (46.0)
Dominant hand, n (%)			
Right side	29 (93.6)	28 (87.5)	57 (90.5)
Painful shoulder, n (%)			
Right side	14 (45.2)	17 (53.1)	31 (49.2)
Duration of shoulder pain (month)⁎⁎	12 (3–24)	18 (6–42)	12 (4–32)
≥ 3 months, n (%)	28 (90.3)	30 (93.8)	58 (92.1)
< 3 months, n (%)	3 (9.7)	2 (6.2)	5 (7.9)
Current medication/treatment status, n (%)			
No treatment	25 (80.7)	23 (71.9)	48 (76.2)
Analgesics	2 (6.5)	1 (3.1)	3 (4.8)
Physical therapy	3 (9.7)	4 (12.5)	7 (11.1)
Others	0 (0.0)	2 (6.3)	2 (3.2)
Physical therapy and analgesics	0 (0.0)	1 (3.1)	1 (1.6)
Analgesics and others	1 (3.2)	1 (3.1)	2 (3.2)
Michigan Body Map (number of pain sites)	2.9 (1.9)	2.3 (1.2)	2.6 (1.6)
Brief pain inventory- short form (BPI-SF)			
BPI-SF pain severity	2.8 (1.6)	2.6 (1.4)	2.7 (1.5)
BPI-SF pain interference	1.8 (1.2)	1.7 (1.3)	1.7 (1.2)
Patient-specific functional scale	4.3 (2.0)	4.2 (1.9)	4.2 (1.9)
Shoulder pain and disability index			
Total score	38.7 (19.1)	33.4 (17.6)	36.0 (18.4)
Pain score	49.2 (18.3)	44.7 (19.9)	46.9 (19.1)
Disability score	32.2 (21.1)	26.4 (17.7)	29.3 (19.5)
Depression, anxiety and stress scale⁎⁎			
Total score	7 (3–13)	7.5 (3–14)	7 (3–13)
Depression score	1 (0–3)	1 (0–3)	1 (0–3)
Anxiety score	2 (0–3)	2 (1–4.5)	2 (1–4)
Stress score	4 (2–7)	3.5 (1.5–6)	4 (2–7)
Pain catastrophizing scale⁎⁎			
Total score	7 (2–11)	9 (4–14.5)	9 (3–14)
Rumination score	3 (0–4)	3 (0.5–6)	3 (0–4)
Magnification score	2 (0–3)	2 (1–3)	2 (0–3)
Helplessness score	3 (1–6)	3 (1–6)	3 (1–6)
Fear-avoidance behaviour questionnaire (FABQ)			
FABQ-work	13.3 (6.1)	14.7 (5.0)	14.0 (5.6)
FABQ-physical activity	9.1 (7.6)	10.5 (8.1)	9.8 (7.8)
Pain self-efficacy⁎⁎	10 (10–12)	11 (10–12)	11 (10–12)
EQ-5D-5L	0.78 (0.10)	0.77 (0.17)	0.77 (0.14)
EQ-VAS	77.9 (14.0)	73.5 (14.9)	75.7 (14.5)
Patient's expectation	11.1 (3.7)	11.8 (3.3)	11.4 (3.5)

⁎ Self-identified ethnicity is categorised according to the Ministry of Health Ethnicity Data Protocols; a participant can be classified as belonging to multiple ethnic groups; therefore, the total percentage does not equate to 100%.

⁎⁎ expressed as median (interquartile range). EQ-5D-5 L, The EuroQol five Dimension five Level; EQ-VAS, The EuroQol visual analogue scale.

Two participants in the sham MWM group refused to receive the intervention on the intervention day due to unforeseen reasons and therefore dropped out of the study accordingly. The angular onset of pain and pain intensity data were not recorded for one participant immediately after the 1^st^ set of 10 repetitions of sham MWM. One participant did not record BPI-SF pain intensity at day 7 follow-up. Within the MWM group, one participant did not complete data for follow-up on day 2, two participants did not complete follow-up on day 3, and one participant did not complete follow-up on day 5.

### Primary outcome

**Fig. 3:**
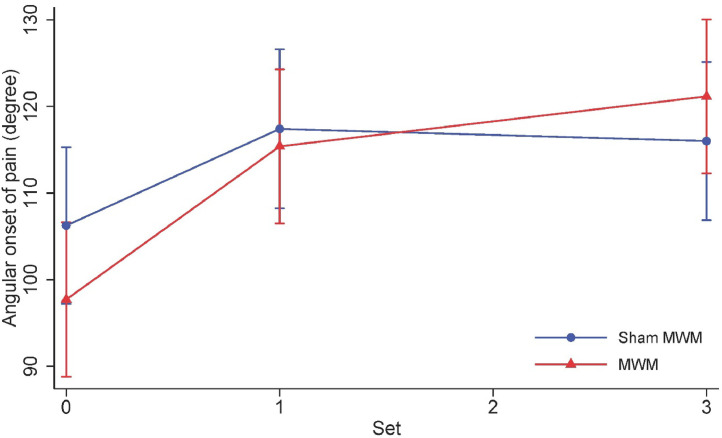
Angular onset of pain at baseline, immediately after receiving 1^st^ set and 3^rd^ set of 10 repetitions of intervention.

The changes in the angular onset of pain for both groups are presented in Fig. 3 and Table 2. Both groups presented an increase in the angular onset of pain after receiving the 1^st^ set of 10 repetitions of intervention (i.e., MWM or sham MWM). After that, participants on the MWM continued to improve, while participants in the sham group presented a slight reduction in the angular onset of pain. The mean and SD values for each group at each time point are presented in Table 2.

**Table 2:** Mean (SD) and between-group estimated mean differences in change in pain intensity, range of motion, pressure pain threshold, and mechanical temporal summation.

Outcome	Sham MWM			MWM			Between-group differences, (95% CI)	
	Baseline	Follow-up 1^st^ set, 10 reps	Follow-up 3 sets, 10 reps	Baseline	Follow-up 1^st^ set, 10 reps	Follow-up 3 sets, 10 reps	Follow-up 1^st^ set, 10 reps	Follow-up 3 sets, 10 reps
Angular onset of pain (degree)	106.3 (22.7)	118.0 (21.5)	116.9 (24.0)	97.7 (26.2)	115.4 (28.4)	121.2 (30.2)	6.5 (−0.9, 13.9)	13.7 (6.3, 21.1)
Pain intensity during shoulder abduction to onset of pain (NRS 0 to 10)	3.5 (1.7)	3.3 (1.9)	3.3 (1.9)	3.1 (1.7)	3.1 (2.0)	2.8 (1.9)	0.1 (−0.4, 0.6)	−0.2 (−0.7, 0.3)
Maximum ROM (degree)	135.6 (23.1)	–	137.7 (25.7)	138.7 (22.3)	–	141.4 (23.7)	–	1.9 (−3.1, 6.8)
Pain intensity during shoulder abduction to the maximum range (NRS 0 to 10)	4.2 (1.9)	–	3.6 (2.0)	3.9 (1.7)	–	3.0 (2.2)	–	−0.3 (−0.9, 0.3)
Pain intensity at rest	1.5 (1.6)	1.5 (1.8)	1.7 (1.7)	1.2 (1.3)	1.5 (1.7)	1.5 (1.6)	0.3 (−0.19, 0.9)	0.1 (−0.4, 0.7)
PPT at shoulder (kPa)	333.3 (133.8)	–	340.9 (135.7)	315.2 (190.0)	–	341.9 (181.5)	–	13.09 (−14.8, 40.9)
PPT at leg (kPa)	479.5 (144.0)	–	498.7 (140.1)	463.46 (190.8)	–	502.97 (195.0)	–	18.9 (−14.6, 52.5)
MTS at shoulder	1.8 (1.5)	–	1.7 (1.4)	2.0 (1.3)	–	1.9 (1.5)	–	−0.1 (−0.6, 0.3)
MTS at leg	1.9 (1.8)	–	1.8 (1.4)	2.0 (1.3)	–	1.9 (1.6)	–	0.0 (−0.5, 0.4)

CI, confidence interval; MTS, mechanical temporal summation; MWM, mobilization with movement; NRS, numeric rating scale; PPT, pressure pain threshold; reps, repetitions; ROM, range of motion.

On average, participants in the MWM group presented an improvement of 6.5° (95% CI −0.9, 13.9) in the angular onset of pain after receiving the 1^st^ set of 10 repetitions compared with the sham MWM group, although the point estimate of 6.5° may not be clinically important. After receiving an additional 2 sets of 10 repetitions of MWM, participants in the MWM group presented an additional improvement of 7.2° (95% CI −0.3, 14.6) in the angular onset of pain when compared with the sham MWM group (Fig. 3 and Supplementary material: Table S1). The between-group difference in angular onset of pain from baseline to after receiving 3 sets of 10 repetitions was 13.7° (95% CI 6.3, 21.1).

### Secondary outcomes

No improvements were observed in pain intensity at rest, pain intensity during shoulder abduction to the onset of pain, maximum ROM, pain intensity during shoulder abduction to the maximum range, PPT, and MTS for both groups from baseline to after receiving the 1^st^ or 3^rd^ set of 10 repetitions of intervention (Table 2). The GROC score was 1.1 (95% CI 0.4, 1.8) higher in the MWM group than in the sham MWM group immediately after receiving the 3^rd^ set of 10 repetitions of interventions (Supplementary material: Table S2). However, no difference was found in the GROC score between MWM and sham MWM groups at follow-up on day 3. Improvements in BPI-SF pain intensity and pain interference from baseline to follow-ups on days 1, 3, 5, and 7 after interventions in the MWM group were not superior to those measures in the sham MWM group, respectively (Supplementary material: Table S3 and Figure S2). We summarized the harms reported by participants using narrative summary. Eight participants reported minor harm at follow-up on day 2. An equal number of harms was reported for the MWM and sham MWM groups (Supplementary material: Table S4).

## Discussion

We assessed the immediate effects of MWM on the angular onset of pain and pain intensity during movement after 1 set and 3 sets of 10 repetitions of interventions. We also explored the incremental effect of dosage of MWM on the angular onset of pain and pain intensity during shoulder abduction. Our results suggest MWM is likely to be effective and that 3 sets of 10 repetitions are required to improve the angular onset of pain in patients with RCRSP.

The between-group difference in angular onset of pain after only 1 set of 10 repetitions was small and not significant (6.5°, 95% CI −0.9, 13.9), suggesting a small benefit for the MWM group. After 3 sets of 10 repetitions, the between-group difference was 13.7° (95% CI 6.3, 21.1), suggesting a greater improvement for patients receiving MWM when compared with sham MWM. We considered the difference in the angular onset of pain (i.e., 13.7°) between interventions as an important finding. Although the estimated effect was smaller than the threshold used for estimating the sample size (i.e., 14.5°), it is still a reasonably large improvement, given the short duration of the intervention. There is no agreement in the literature regarding what is clinically meaningful for the angular onset of pain measurement. Some literature suggests shoulder abduction increment of 10° is a clinically important improvement.^15^ The observed incremental effect of 7.2° (95% CI −0.3, 14.6) suggests a likely improvement for the MWM group, given that improvements of up to 14.6° is reasonably compatible with our data. Together, those point estimates and confidence intervals indicate a likely benefit of receiving two further sets. Participants in the sham MWM group presented a slight decrease in the angular onset of pain as the 2^nd^ and 3^rd^ sets of interventions were delivered.

To date, there was conflicting evidence that MWM improves the angular onset of pain in patients with RCRSP.^5^, ^6^, ^7^, ^8^ Previous trials had limitations such as using crossover study designs with relatively small sample size and lack of participants’ clinical characteristics. The sample size in those studies ranged from 24 to 42, including a study^8^ without sample size calculation and a study^6^ with an inappropriate sample size calculation. Our trial addressed the limitations raised by previous systematic reviews with a proper sample size calculation conducted in the design stage and is one of the largest trials that has been conducted for assessing the effects of MWM on shoulder ROM and pain intensity in patients with RCRSP to the best of our knowledge.^4^^,^^39^

In practice, clinicians would generally discard the MWM technique immediately if there is no positive response on the initial assessment and select other techniques.^1^^,^^2^ Previous trials may have underestimated the effect of MWM as they seem to have not screened participants for an immediate positive response to the technique prior to delivering the intervention.^5^, ^6^, ^7^, ^8^ We followed recommendations from the literature^1^^,^^2^ and only included participants who responded positively to MWM. Our findings can be generalized to this specific targeted population by excluding those who do not positively respond to MWM on the initial assessment in clinical setting.^40^^,^^41^ Hence, our study has good external validity and reflects clinicians who would discard this technique immediately if there is no positive response on initial assessment and select other techniques.^1^^,^^2^

Our findings suggest that the 3 sets of 10 repetitions of MWM intervention did not improve pain intensity during shoulder abduction to the onset of pain. There is conflicting evidence that MWM improves pain intensity during shoulder abduction to the onset of pain in patients with RCRSP.^5^^,^^7^ The inconsistent findings could be due to different numbers of MWM treatment sessions tested within those trials. Our study and the one by Ribeiro et al.^7^ only offered one treatment session to participants. In contrast, participants received four treatment sessions in the study by Delgado-Gil et al.^5^ It is possible that more treatment sessions are needed to reveal changes in pain intensity during movement between the two interventions.

The between-group difference in the GROC scores was lower than the minimal clinically important difference for that outcome.^42^ This could be explained by the fact that participants in the MWM group only improved their angular onset of pain, but they presented no change in pain at rest and pain intensity during shoulder abduction to the onset of pain and maximum ROM. Together these findings suggest that one session of MWM intervention is not enough to improve maximum ROM, pain intensity during movement, and successful outcome on the GROC score, and more treatment sessions or other forms of interventions may be required.

Our findings suggest that 3 sets of 10 repetitions of MWM did not effectively improve pain intensity and pain interference over time at follow-ups. To date, only one study^15^ explored short-term effects of one session of MWM on rest pain and angular onset of pain in patients with RCRSP and found that one session of MWM immediately improved rest pain and angular onset of pain. Such improvement lasted up to 30 minutes but was not sustained longer than that over a 7-day follow-up.^15^

MWM did not change PPT and MTS when compared with sham MWM. These findings are consistent with those from a previous study, which showed improvements in the angular onset of pain were unrelated to PPT changes.^8^ Together, these findings provide preliminary evidence that improvement in the angular onset of pain may not be related to modulation of hyperalgesia (assessed through PPT) and summation of nociceptive inputs (assessed through the MTS test). However, improvements in the angular onset of pain could have been elicited by cortical mechanisms and supraspinal mediated descending modulation of nociceptive inputs,^43^ which were not measured in this trial. The exact mechanism through which MWM improves the angular onset of pain is yet to be determined.

There are some limitations in this study. Firstly, we only assessed the immediate and short-term effects of MWM versus sham MWM on clinical outcomes in patients with RCRSP. Secondly, we only assessed the isolated effect of posterolateral glide MWM on the glenohumeral joint and did not assess the effect of MWM interventions on other joints (e.g., scapulothoracic and thoracic region joints) or the effect of MWM when combined with other modalities (e.g., advice, exercise therapy). Thirdly, participants in this study presented low average scores for psychological outcome measures, indicating that this sample did not present psychological distress related to their shoulder pain. This occurred by chance but does impact on the external validity of our study. Our findings may not be generalizable to patients presenting with shoulder pain and experiencing high level of psychological distress. Lastly, we recruited participants with acute and chronic shoulder pain, but most participants (*n* = 58) presented with chronic shoulder pain. The course and prognosis of shoulder pain differs between patients with acute and chronic shoulder pain.^44^ This suggests the effect of MWM on clinical and quantitative sensory testing outcomes may differ between patients with acute and chronic shoulder pain. Our findings could not be applicable to patients presented with acute shoulder pain.

## Conclusion

Three sets of 10 repetitions of MWM improved the angular onset of pain but not pain intensity in patients with RCRSP. One set of 10 repetitions of MWM caused small improvements in the angular onset of pain, however, the improvement is unlikely to be clinically relevant. The findings of this study provide preliminary support for the use of MWM in treating patients with RCRSP and provide some guidance for clinicians to decide the MWM dosage to be used in clinical practice.

## Conflicts of interest

The authors declares no conflicts of interest.

## Appendix Supplementary materials

Image, application 1
