# Supplementary material for: The immediate effects of mobilization with movement on shoulder range of motion and pain in patients with rotator cuff-related shoulder pain: A randomized controlled trial (Evolution Trial)
Source: Braz J Phys Ther. 2024 Nov 20;28(6):101145. doi: 10.1016/j.bjpt.2024.101145 (PMC11724996; doi:10.1016/j.bjpt.2024.101145)
Supplement: Supplementary file 1 [file mmc1.pdf]

Supplementary online material

Table S1. Incremental effects of MWM on the angular onset of pain and pain intensity at the onset of pain during shoulder abduction.

**Outcome**	**Sham MWM**		**MWM**		**Between-group difference (95% CI)**
	**Follow-up 1st set, 10 reps**	**Follow-up 3 sets, 10 reps**	**Follow-up 1st set, 10 reps**	**Follow-up 3 sets, 10 reps**	**Follow-up 3 sets, 10 reps**
**Angular onset of pain (degree)**	118.0 (21.5)	116.9 (24.0)	115.4 (28.4)	121.2 (30.2)	7.2 (-0.3, 14.6)
**Pain intensity (NRS 0 to 10)**	3.3 (1.9)	3.3 (1.9)	3.1 (2.0)	2.8 (1.9)	-0.3 (-0.8, 0.2)

CI, confidence interval; MWM, mobilisation with movement; NRS, numeric pain rating scale; reps, repetitions.

Table S2. Global rating of change scale immediately after receiving 3 sets of 10 repetitions and follow-up on day 3 (mean and standard deviation).

Time point	Sham MWM group	MWM group	Between-group difference (95% CI)
**Immediately after 3 sets**	0.5 (1.5)	1.6 (1.3)	1.1 (0.4, 1.8)
**Day 3 after intervention**	0.6 (1.1)	1.0 (1.3)	0.4 (-0.2, 1.1)

MWM, mobilisation with movement

Table S3. Brief Pain Inventory-Short Form at baseline and follow-ups after intervention (mean and standard deviation).

Outcome	Sham MWM	MWM	Between-group difference in change (95% CI)
**BPI-pain intensity**			
**Baseline**	2.8 (1.6)	2.6 (1.4)	--
**Day 1**	2.1 (1.2)	2.1 (1.3)	0.2 (-0.4, 0.7)
**Day 3**	1.9 (1.4)	1.8 (1.1)	0.1 (-0.4, 0.7)
**Day 5**	1.8 (1.3)	2.0 (1.3)	0.4 (-0.2, 0.9)
**BPI-pain interference**			
**Baseline**	1.8 (1.2)	1.7 (1.3)	--
**Day 1**	1.0 (0.8)	1.1 (0.8)	0.0 (-0.5, 0.5)
**Day 3**	0.9 (0.8)	1.0 (1.0)	0.1 (-0.4, 0.5)
**Day 5**	0.9 (1.1)	1.1 (1.3)	0.1 (-0.4, 0.6)
**Day 7**	1.0 (1.0)	0.9 (0.8)	-0.2 (-0.6, 0.3)

Data are mean and standard deviation. BPI-SF, Brief Pain Inventory-Short Form; CI, confidence interval; MWM, mobilisation with movement; SD, standard deviation.

Table S4. Reported adverse event in sham MWM and MWM groups at follow-up on day 2.

**Intervention**	Adverse events
**MWM group**	The overuse of the shoulder.
	The intervention aggravated the pain in the biceps region.
	Pain was greater than usual after intervention.
	Soreness in the neck after the intervention.
**Sham MWM group**	Increased soreness after the intervention.
	Pain at rest slightly increased than usual.
	Painful ache that lasted for most of the day after the intervention.
	Shoulder pain increased after the intervention.

MWM, mobilisation with movement

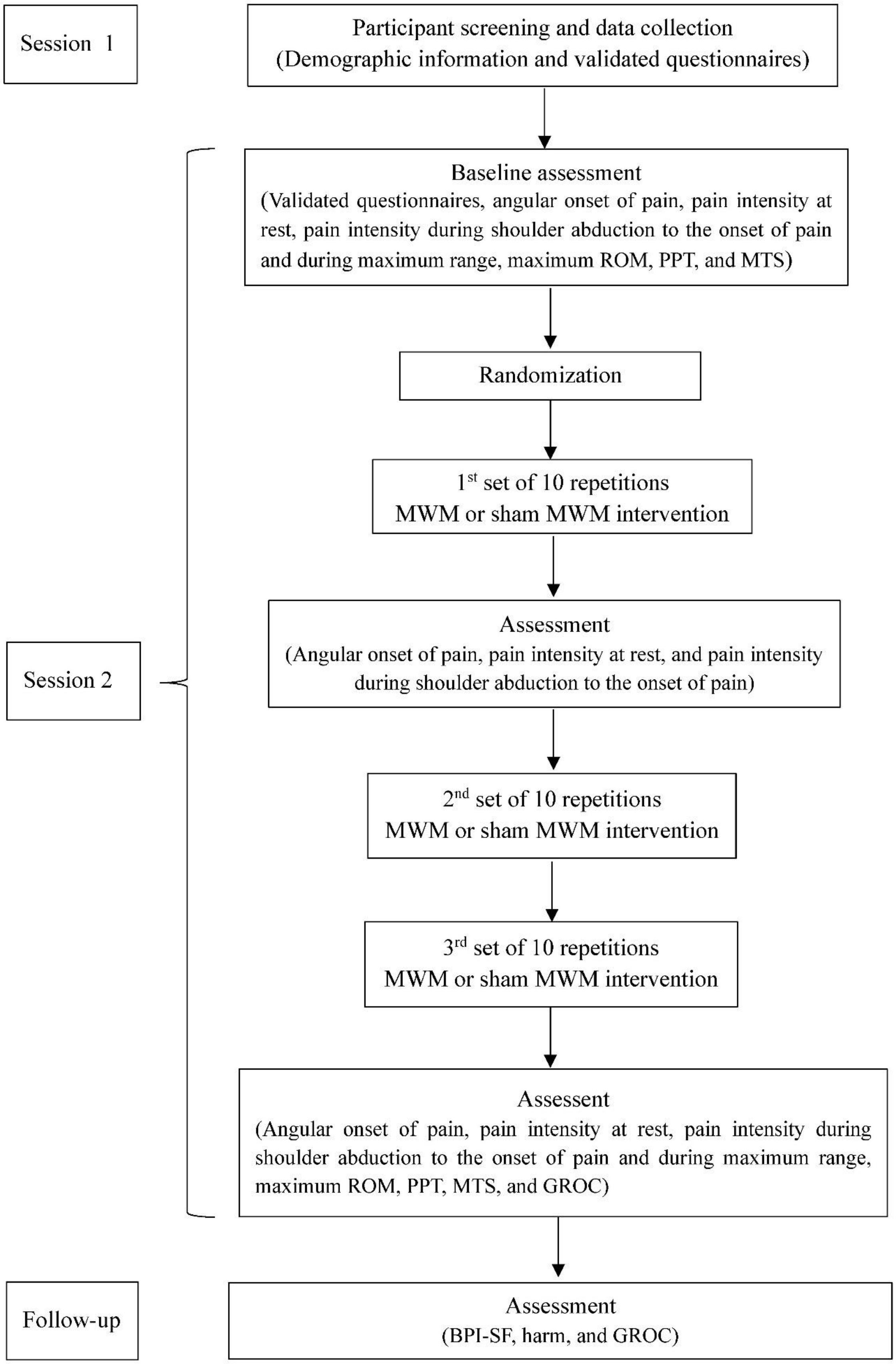

Figure 1S. Flowchart of procedure.

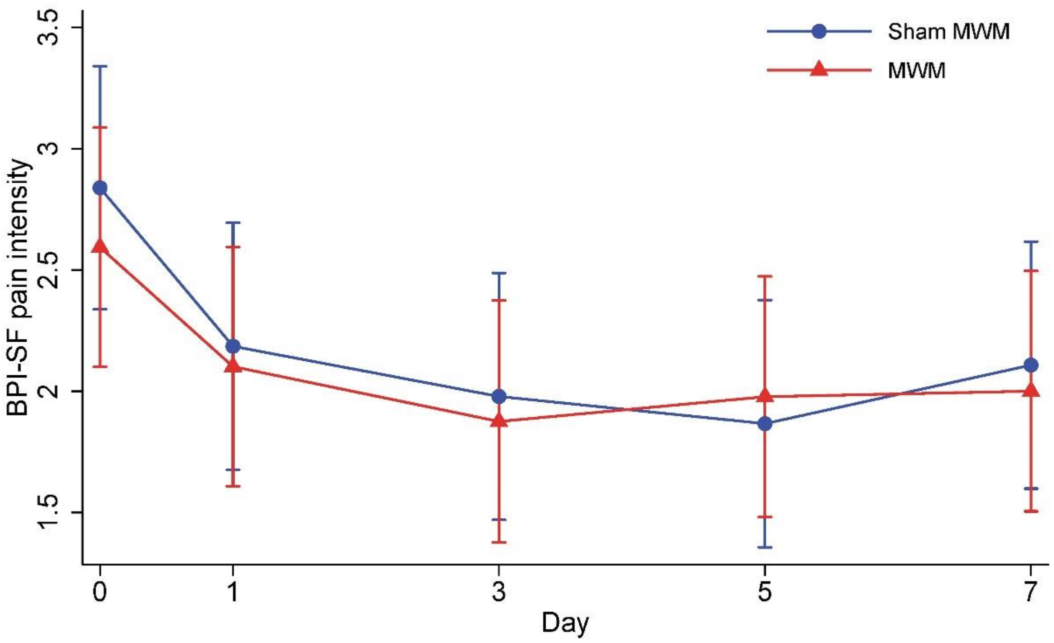

(A)

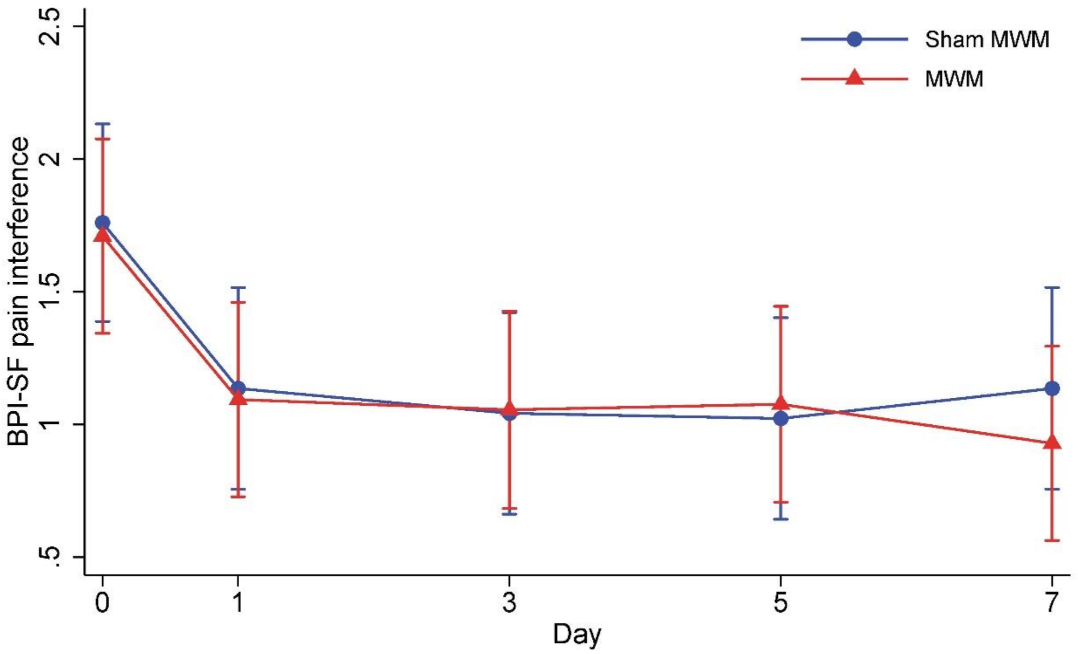

(B)

Figure S2. Brief Pain Inventory-Short Form (A) pain intensity and (B) pain interference at baseline and follow-ups (days 1, 3, 5, and 7). MWM, mobilisation with movement
